# Qualitative and quantitative phytochemical screening of *Nerium oleander* L. extracts associated with toxicity profile

**DOI:** 10.1038/s41598-022-26087-0

**Published:** 2022-12-11

**Authors:** Neşe Bakir Çilesizoğlu, Emine Yalçin, Kültiğin Çavuşoğlu, Selin Sipahi Kuloğlu

**Affiliations:** 1 Department of Biology, Institute of Science, Giresun University, Giresun, Turkey; 2 Department of Biology, Faculty of Science and Art, Giresun University, 28200 Giresun, Turkey

**Keywords:** Biochemistry, Genetics, Physiology

## Abstract

In this study, phytochemical analysis and toxicity profile of leaf and flower extracts of *Nerium oleander* L. species collected from Giresun province (Turkey) were investigated. In phytochemical analyzes, the cardiac glycoside, alkaloid, saponin and tannin contents of the extracts were analyzed qualitatively and quantitatively. The physiological effects of extracts were determined by examining root elongation, weight gain and germination rates. Biochemical effects were determined by measuring the levels of malondialdehyde (MDA), glutathione (GSH), superoxide dismutase (SOD) and catalase (CAT), which are indicators of oxidative stress. Cytotoxic and genotoxic effects were investigated by mitotic index (MI), micronucleus (MN) and chromosomal abnormality (CA) tests. *N. oleander* leaf and flower extract applications caused significant decreases in the physiological parameters of *Allium* bulbs. SOD and CAT activity in root tip cells increased significantly after the application of leaf extract compared to the control group. Similar changes were observed in the application of flower extract, but these increases were found to be at a lower level compared to the increases induced by the leaf extract. An increase in MDA levels and a decrease in GSH levels were observed in groups treated with leaf and flower extracts. These changes show that the extracts cause deterioration in antioxidant/oxidant balance. It was determined that the extracts, which caused a decrease in MI rates and an increase in MN and CAs frequencies, showed the most prominent cytotoxic and genotoxic effects at 250 μg/mL doses. These toxic effects were associated with the phytochemical content of the extracts, and it was thought that cardiac glycosides and alkaloids, whose presence were detected in qualitative and quantitative analyzes, may play an important role in toxicity. Studies investigating the therapeutic properties of plants as well as their toxic effects are insufficient, which leads to the fact that plants exhibiting potential toxicity are not well known. Therefore, this study will lead many studies on the toxicity profile of the phytochemical contents of plants. Therefore, this study will draw attention to the investigation of the toxicity profile and phytochemical contents of plants and will lead to similar studies.

## Introduction

Plants are an important source of a wide variety of secondary metabolites used in the treatment and prevention of diseases^1^. The pharmacological and biological effects of many plants have been elucidated by isolating and characterizing the active ingredients^2^. Today, these active ingredients are also used as pharmaceutical raw materials^3^. The vast majority of drugs are produced from herbal secondary metabolites and compounds derived from these metabolites^4,5^. Secondary metabolites that ensure the survival of the plant have many activities such as antioxidant, antiviral, antimalarial, analgesic, diuretic, anthelmintic, antibacterial, anticancer, antiinflammatory, antifungal, antiallergic and antimutagenic^6^. There are many studies in the literature reporting the protective properties of herbal extracts and compounds isolated from plants against many diseases. The toxic properties of many plant species whose protective properties against many diseases are determined are not investigated and such properties remain in the background. The toxicity of plants especially those consumed as food and used in houses, parks, gardens and various landscaping applications should be investigated. Although there are many literature studies investigating the protective properties of herbal extracts against various damages, studies investigating their toxic properties are not yet at the desired level. In this study, the toxic effects of *Nerium oleander* L., which has been reported to have a protective effect against cancer in literature, were investigated.

*Nerium oleander* is a species that contains a high variety of bioactive compounds and has antioxidant, anticancer, antimicrobial and antidiabetic activities^7,8^. *N. oleander* is a shrub or small tree form, generally found in warm and subtropical climates, belonging to the Apocyanaceae family and the *Nerium* genus^9^. *N. oleander* exhibits many biological and pharmacological activities with its rich phytochemical content. *Nerium* leaves are rich in carbohydrates, flavonoids, alkaloids, steroids, cardiac glycosides and tannins^10^. Among the polyphenols contained in the leaf, there are high levels of cinnamic acid and small amounts of epicatechin, catechin and chlorogenic acid. In addition to phenolic compounds, the leaves contain 2.3% crude polysaccharides and arabinose, galacturonic acid, galactose and rhamnose are the important polysaccharides detected^11^. *N. oleander* flowers are rich in phenol, tannin, flavonoid, coumarin, alkaloid, phlobatannin, triterpene^12^. There are various studies on the phytochemical content and biological activities of *N. oleander* in the literature. Yamauchi et al.^13^ reported the antibacterial activity of root bark tissue of *N. oleander* containing active components such as odoroside B and oleandrigenin. Luay et al.^14^ reported that *N. oleander*, which contains the 3β,14β-dihydroxy-5β-card-20(22)-enolide structure, exhibited significant anticancer activity. Nurgun et al.^15^ reported that ethanolic extracts of fresh and dried flowers of *N. oleander* exhibited antinociceptive and anti-inflammatory activity in mice. Siddiqui et al.^16^ reported that compounds such as diginose and oleandrose isolated from *N. oleander leaves* exhibited potent cytotoxic effects in many cell lines tested. Hovhanissyan et al.^17^ determined that *N. oleander* extract induced apoptosis in the cells and exhibited a cytotoxic effect. It is also reported in the literature that the oleandrin contained in *N. oleander* extract is a cytotoxic agent, induces the formation of reactive oxygen species and increases apoptosis and cell damage in this way^5^.

There are many studies in the literature investigating the phytochemical content and biological properties of plants, but the high diversity of plants in the world and Turkiye makes these studies insufficient^18^. In addition, plants produce different secondary compounds in order to adapt to the environment and ecological conditions in which they grow^19^. In this respect, the phytochemical content and biological effect of the same species grown in different regions also vary. In this study, leaf and flower parts of *N. oleander* collected from Giresun province were extracted, phytochemical analysis and toxic effects were investigated. Toxic effects were associated with the phytochemical content determined by qualitative and quantitative analysis. The physiological, cytotoxic, genotoxic, biochemical and anatomical effects of *N. oleander* leaf and flower extracts were investigated in *Allium assay,* which is a bioindicator test^20^. *Allium* test is a fast, easy-to-apply and low-cost method among toxicity tests. In recent years, *Allium* test has been widely used to determine the toxic effects of herbal extracts^21,22^. The results obtained with this test show high compatibility with other toxicity tests. A high correlation was obtained between the results of the cytotoxicity test applied to human lymphocyte cells and algal cells and the results of the *Allium* test^23^. In similar studies, genotoxicity test in rodents and *Allium* test results show 82% similarity. In the literature, it was emphasized that this test was highly acceptable in the evaluation of the cytotoxic and genotoxic effects of chemicals, and the same results were obtained with the toxicity test results performed in the bone marrow of Wistar rats^24,25^. In this study, the phytochemical content of *N. oleander*, which is widely distributed in Giresun province, and a comprehensive toxicity profile on *Allium cepa* were investigated.

## Materials and methods

### Collection and extraction of samples

**Figure 1:**
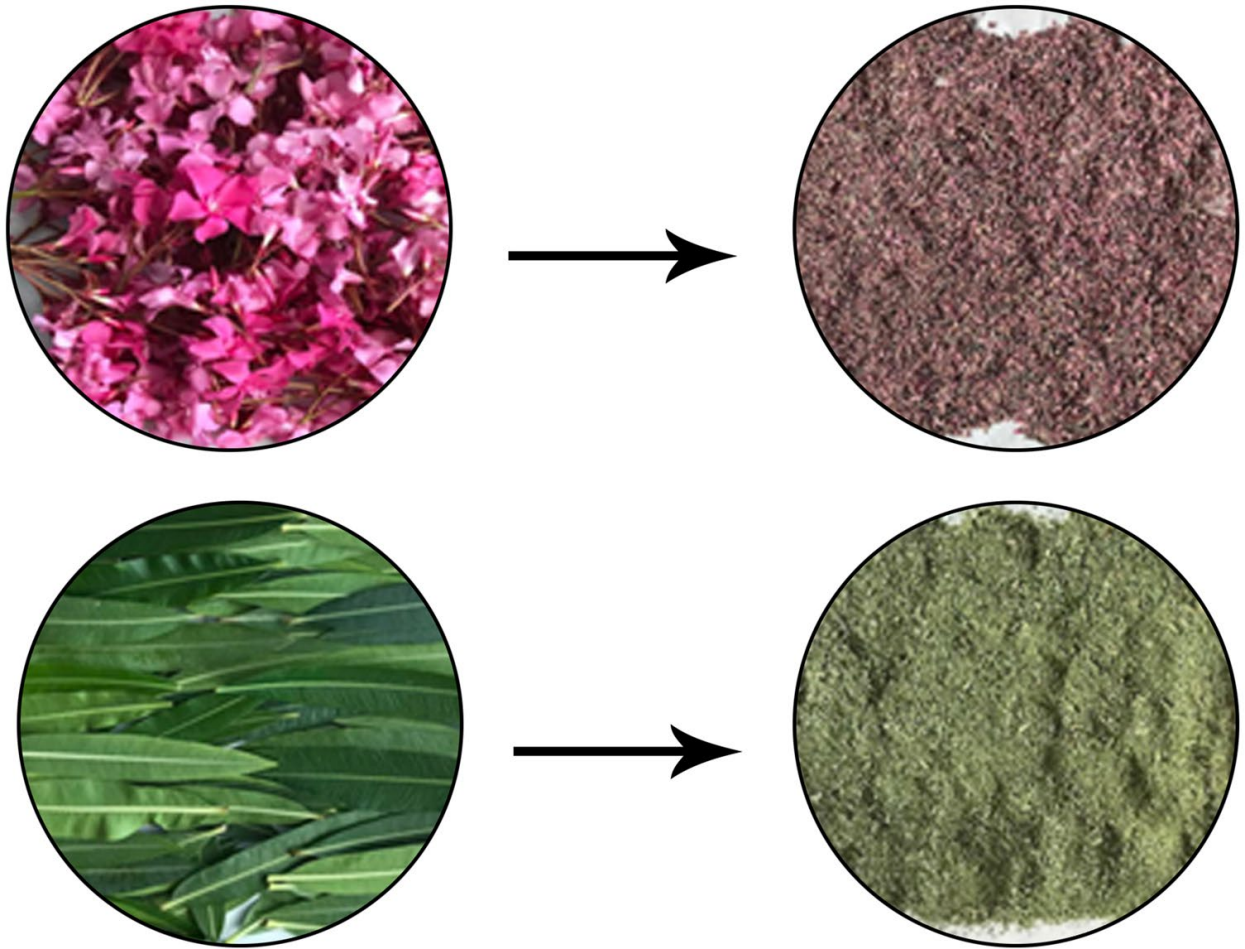
Appearance of *N. oleander* leaf and flower tissues before and after drying.

Flower and leaf parts of *N. oleander* were collected from Giresun University-Güre location (40° 54′ 52.2″ N; 38° 19′ 27.7″ E) in August-2021 and identified in Giresun University Botany Department. A voucher specimen is deposited in the Herbarium of the Biology Department in Giresun Univesity with a voucher number BIO-Neol1224/2021. The homogeneous, disease-free and non-pale parts were dried in the oven at 40 °C for 4–5 days and then ground into powder using a grinder (Fig. 1.). Leaf and flower tissues were extracted by maceration method using two different solvents, water and methanol. 5 g of leaf and flower powder were extracted separately in 100 mL of water and methanol solvents in a shaking incubator for 12 h at room temperature and then filtered with Whatman No: 4 filter paper. At the end of the incubation period, the solid particles were filtered off and the resulting filtrate was centrifuged at 10,000 rpm for 10 min. The supernatant was evaporated with an evaporator until a dry pellet was obtained and the resulting pellet was used for further analysis. Evaporation of solvents was carried out under reduced pressure at 40 °C to avoid damage to the active compounds by heating. *N. oleander* leaf and flower extracts thus obtained were coded as Noex-I and Noex-II, respectively. On average, 0.34 g (6.9%) crude extract was obtained in the methanol extraction and 0.42 g (8.4%) crude extract was obtained in the water extraction of the flower. In leaf extraction; crude extract for water and metanol were 0.51 g (10.2%) and 0.45 g (%9), respectively.

Experimental research on plant samples, including the supply of plant material, complies with institutional, national and international guidelines and legislation.

### Qualitative phytochemical analysis

Phytochemical analysis of *N. oleander* leaf and flower extracts was carried out by qualitative methods and cardiac glycoside, alkaloid, saponin and tannin contents of the *N. oleander* leaf and flower tissues were investigated.

### Test for cardiac glycoside

Qualitative analysis of cardiac glycosides in the extracts was performed with the Keller Killiani test. 1 mL of acetic acid and 2 drops of ferric chloride were added to 2 mL of extract, then 2 mL of sulfuric acid (concentrated) was added and the color change was observed. Reddish-brown color formation was deemed to be a positive test for cardiac glycosides^26,27^.

### Test for alkaloid

For qualitative alkaloid determination, a few drops of Meyer's reagent were added to 1 mL of extract. The formation of a creamy white precipitate was considered positive for the alkaloid^26^.

### Test for saponin

To detect the presence of saponin, 5 mL of distilled water was added to 1 mL of extract and vortexed for 10 min. The formation of a foam column that did not disappear with the addition of HCl was evaluated as positive for saponin^27,28^.

### Test for tannin

For qualitative tannin analysis, a few drops of lead acetate were added to 1 mL of extract. A large white-brown precipitate formation was considered a positive test for tannin^26^.

### Quantitative phytochemical analysis

Quantitative analysis of phytochemical components, whose presence was tested by qualitative analysis, was also carried out.

### Cardiac glycoside determination

Cardiac glycoside analysis was determined according to the method reported by Tofighi et al.^29^. 10 g of *N. oleander* leaf and flower extracts were mixed with 10 mL of Baljet's reagent. After 1 h of incubation, 20 mL of distilled water was added and the absorbance was measured at 495 nm. Securidaside was used as a standard, and the amount of cardiac glycoside was expressed as mg securidaside equivalent (SE)/g.

### Tannin determination

Quantitative tannin content in the extracts was tested according to the method suggested by Mital and Jha^30^. 1 mL of extract was mixed with 0.5 mL of Folin reagent, and then it was saturated with 1 mL of Na_2_CO_3_ and 8 mL of distilled water was added to the final mixture. The solution, which was incubated for 30 min, was centrifuged and the supernatant was analyzed at 725 nm. Tannic acid was used as a standard and tannin content was expressed as mg tannic acid equivalent (TAE)/g.

### Total saponin determination

The determination of the total saponin content in the extracts was made according to the vanillin-sulfuric acid colorimetric method^31^. The mixture containing 250 µL of vanillin reagent, 50 µL of extract and 2.5 mL of 72% sulfuric acid was mixed and incubated at 60 °C for 10 min. At the end of the incubation, the absorbance of the solution cooled in an ice bath was read at 544 nm. Saponin content was expressed as mg diosgenin equivalent (DE)/g.

### Alkaloid determination

Alkaloid determination was made according to the method reported by Selvakumar et al^32^. 2 mL of HCl was added to the extract dissolved in dimethylsulfoxide and the mixture was filtered. An equal volume of bromocresol green and phosphate buffer was added to the solution. The mixture was shaken by adding chloroform. The absorbance of the solutions containing the extract and atropine used as a standard was read at 470 nm. The alkaloid content was expressed as mg atropine equivalent (AE)/g. All tests were repeated in triplicate. Calibration curves of standards were given in Supplementary Fig. S1.

### Toxicity tests

The toxic effects of *N. oleander* leaf and flower extracts were investigated using the *Allium* test. Untreated *Allium cepa* L. (2n = 16) bulbs with a diameter of 1.5–2.2 cm used in toxicity testing were purchased from a local supermarket and identified in the Botanical department. *A. cepa* bulbs were rinsed with distilled water and their outer scales were removed. Old root remnants were carefully removed to preserve the root primordia. After these preliminary applications, the bulbs were used in toxicity tests. Toxicity was investigated by physiological, biochemical, cytogenetic and anatomical parameters. Seven different groups were formed to investigate the dose-related toxicity of *N. oleander* leaf and flower extracts (Table 1). EC_50_ is defined as the concentration that produces a 50% reduction in root growth. So, inhibitions in *Allium* root growth were examined to determine the EC_50_ value of the extracts. For this purpose, the effects of leaf and flower extract applications in the range of 10–500 μg/mL on root elongation were investigated. EC_50_ value was found 300 μg/mL for leaf extract and 425 μg/mL for flower extract. In order to obtain healthy and sufficient root tissue required in toxicity tests, the doses applied in the experimental groups were selected from the values below the EC_50_ value of both extracts.

**Table 1:** Experimental groups of Noex-I and Noex-II applications.

Groups	Treatments
Group I (control)	Tap water
Group II	50 μg/mL Noex-I
Group III	100 μg/mL Noex-I
Group IV	250 μg/mL Noex-I
Group V	50 μg/mL Noex-II
Group VI	100 μg/mL Noex-II
Group VII	250 μg/mL Noex-II

### Physiological parameters

The effects of *N. oleander* leaf and flower extracts on germination were investigated by root length, weight gain, germination percentage and relative injury rate. For this aim, bulbs were placed in glass beakers and germinated in related solutions in the incubator at 22 °C for 72 h. Ten bulbs were used for each group. The solutions of each group were checked daily. At the end of the germination period, the best-developed 10 roots of each bulb were measured and mean root lengths were calculated. Weight gain was calculated by taking the difference between the initial and final weights of the bulbs. Relative injury rate (RIR) and germination percentage (GP) and were calculated using Eqs. (1) and (2), respectively^33^.

1 $$ {\text{GP }}\left( \% \right): \, \left[ {{\text{Number}}\,{\text{of}}\,{\text{germinated}}\,{\text{bulbs}}} \right]/\left[ {{\text{Total}}\,{\text{number}}\,{\text{of}}\,{\text{bulbs}}} \right] \, \times { 1}00, $$

2 $$ {\text{RIR}} = \, \left[ {{\text{GP}}\% \,{\text{of}}\,{\text{control }}{-}{\text{ GP}}\% \,{\text{of}}\,{\text{treatment}}\,{\text{group}}} \right]/\left[ {{\text{GP}}\% \,{\text{of}}\,{\text{control}}} \right]. $$

### Cytotoxic and genotoxic effects

The changes in the mitotic index (MI), micronucleus (MN) and chromosomal abnormalities (CAs) ratios in the *Allium* test were investigated to determine the cyto- and genotoxic effects of *N. oleander* leaf and flower extracts. For this purpose, 1 cm long samples were collected from each bulb at the end of the germination period and fixed in Clarke solution. After fixation, the samples washed with 96% ethanol were incubated in 1 N HCl for 17 min at 60 °C for hydrolysis. After the completion of all procedures, the root tips were stained with acetocarmine for 24 h and examined under a research microscope^34^. After germination procedure root slides were prepared and mitotic cells were examined. A total of 10.000 cells were counted for each group and MI percentages were calculated using Eq. (3). Cells in prophase, metaphase, anaphase and telophase were taken as basis in determining the number of dividing cells^35^.

3 $$ {\text{MI}}\% \, = \, \left[ {{\text{Number}}\,{\text{of}}\,{\text{ dividing}}\,{\text{cells}}} \right]/\left[ {{\text{Total}}\,{\text{number}}\,{\text{of}}\,{\text{cells}}} \right] \, \times { 1}00. $$

CAs and MN frequencies were investigated in order to determine the genotoxic effects of *N. oleander* leaf and flower extracts. In the detection of MN and CAs 1000 cells were counted for each group.

### Antioxidant/oxidant dynamics

In order to determine the effects of *N. oleander* leaf and flower extracts on antioxidant and oxidant balance, malondialdehyde (MDA), glutathione (GSH), superoxide dismutase (SOD) and catalase (CAT) levels were measured (triplicate) in root tip cells. Root tip samples of each group were first homogenized in phosphate buffer and centrifuged. The obtained supernatant was used in SOD and CAT analysis.

### SOD and CAT levels

In SOD activity measurements, a mixture containing 1.5 mL sodium phosphate buffer, 0.3 mL methionine, 0.3 mL nitroblue tetrazolium chloride, 0.3 mL EDTA-Na_2_, 0.3 mL riboflavin, 0.01 mL extract, 0.01 mL polyvinylpyrrolidone and 0.28 mL of distilled was prepared. The mixtures were placed under a 15 W fluorescent lamp for 10 min and absorbance was measured at 560 nm at the end of the time^36^. For the determination of CAT activity, 0.2 mL of extract was added to the mixture containing 0.3 mL of H_2_O_2_, 1.0 mL of distilled water and 1.5 mL of sodium phosphate buffer. The CAT activity was measured by spectrophotometrically monitoring the decrease in the amount of H_2_O_2_ at 240 nm^37^.

### MDA and GSH levels

For MDA measurement, 0.5 g sample of each group was homogenized in trichloroacetic acid and centrifuged at 12,000 rpm for 15 min, and MDA analysis was performed in the supernatant. A mixture of 5% thiobarbituric acid and supernatant (1:1) was boiled at 96 °C for 25 min. At the end of the time, the cooled mixture was centrifuged at 10,000 rpm for 5 min and the absorbance of the supernatant was measured at 532 nm^38^. For GSH analysis, the samples belonging to each group were shaken with the same volume of 10% trichloroacetic acid in ice for 15 min and centrifuged at 5000 rpm for 15 min. A mixture containing 0.4 mL of supernatant, 0.8 mL of Tris buffer, and 0.02 mL of DTNB was prepared and the absorbance was read spectrophotometrically at 412 nm after waiting for 5 min^39^.

### Anatomical alterations

To determine the anatomical effects of *N. oleander* leaf and flower extracts on *A. cepa* root tip cells, cross-sections were taken from root tip cells. The sections were stained with methylene blue, examined and visualized with a light microscope. The frequency of anatomical damages was determined by preparing 10 preparations from the samples belonging to each group^40^.

### Statistical analysis

Analyzes were performed using the “IBM SPSS Statistics 22” package program and the data obtained were given as mean ± SD (standard deviation). Statistical significance between the means was determined by Duncan's test and One-way ANOVA, and it was considered statistically significant when the p value was < 0.05.

## Results and discussion

In this study, the leaf and flower parts of *N. oleander* samples were collected from Giresun and then the phytochemical analysis and toxic effects were investigated. The phytochemical content was examined by qualitative tests and the toxic effects of the extract were associated with this ingredient.

### Qualitative phytochemical analysis

**Figure 2:**
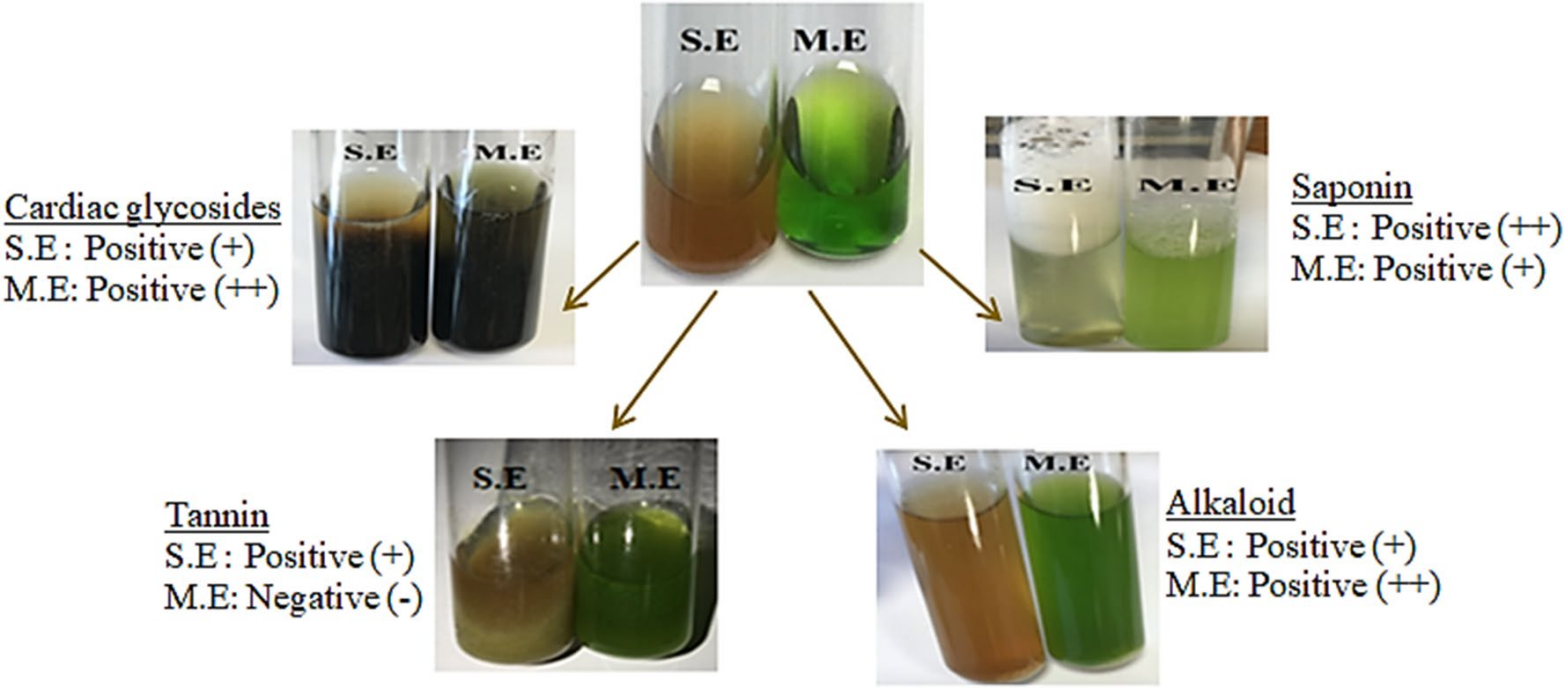
Qualitative analysis of Noex-I. S.E and M.E indicate extraction with water and methanol, respectively.

The samples obtained by water and methanol extracts of *N. oleander* leaf and flowers were used in qualitative-phytochemical analysis. Positive results were obtained in terms of cardiac glycosides, saponins, tannins and alkaloids in *N. oleander* leaf extract except for tannin in methanol extract (Fig. 2). Cardiac glycosides were detected in the extraction with both solvents and it was determined that the cardiac glycoside content was more intense in the methanol extract. While the presence of saponin was detected in both extracts, the fact that a denser foam formation was observed especially in the extract obtained with water, compared to methanol, indicates that the amount of saponin in this extract is higher. In terms of tannin content, water extract was evaluated as positive and methanol extract as negative. A stronger positivity was determined in the methanol extract of alkaloid content, but a positive result was obtained in terms of the presence of alkaloids in both extracts. In the literature, there are studies in which phytochemical analyzes of various parts of *Nerium* species are carried out. Bhuvaneshwari et al.^41^ performed qualitative analyzes with *N. oleander* leaf extracts and obtained positive results in terms of terpenoids, alkaloids, cardiac glycosides, saponins and tannins. Rajendra et al.^42^ detected the presence of phenolic compounds, alkaloids, tannins, flavonoids and cardiac glycosides in the extracts of *N. indicum* leaves obtained with benzene and alcohol extraction, and reported negative results in terms of anthroquinone glycoside and carbohydrate.

**Figure 3:**
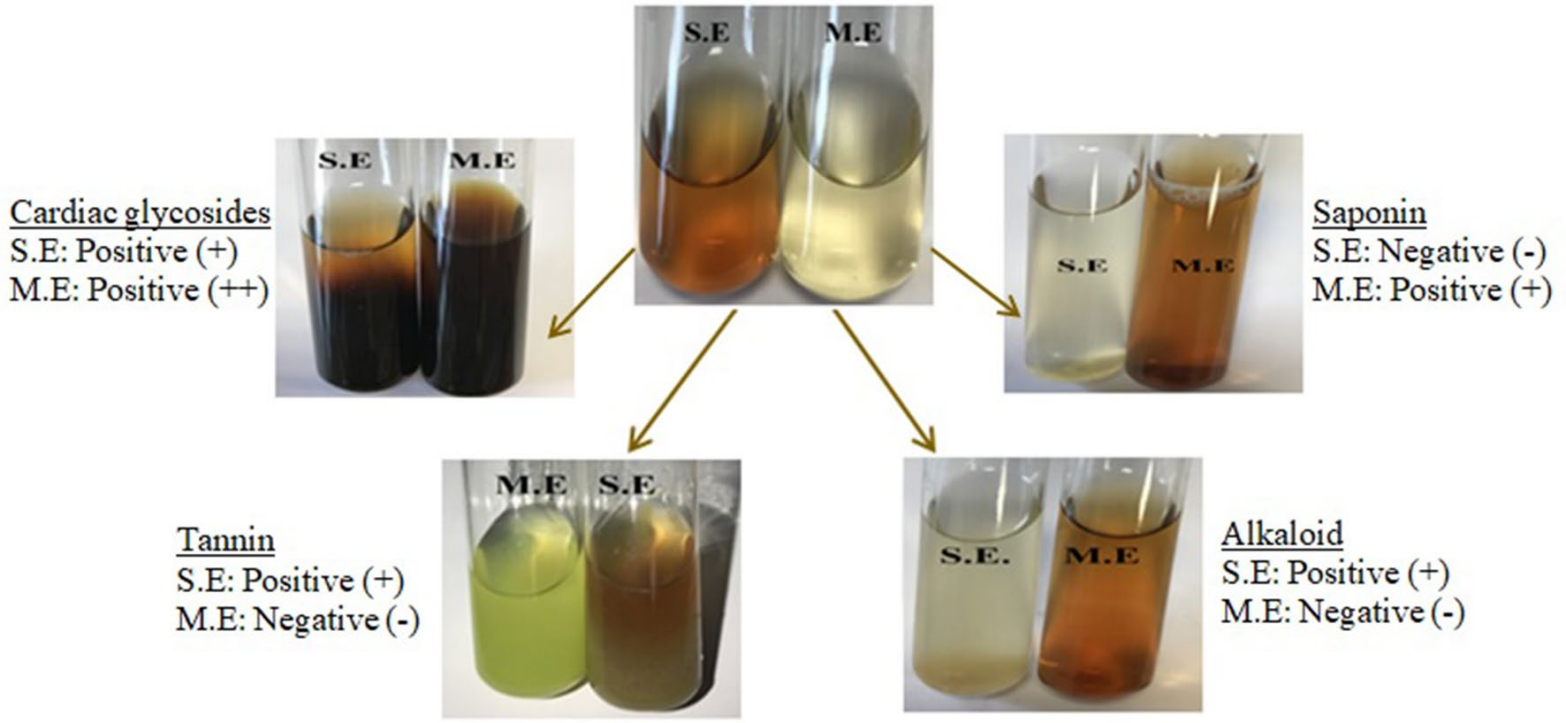
Qualitative analysis of Noex-II. S.E and M.E indicate extraction with water and methanol, respectively.

Flower tissues of *N. oleander* were also analyzed qualitatively by the same phytochemical analyzes and the results are given in Fig. 3. Similar to the leaf extract, cardiac glycosides were detected in both solvents in the flower extract, and the cardiac glycoside content was found to be more intense in the methanol extract. While the presence of saponin was not detected in the aqueous extract, very low foam formation was observed in the methanol extract and this result was evaluated as low saponin presence compared to the leaf extract. While negative results were obtained in methanol extract in terms of tannin and alkaloid, positive results were obtained in aqueous extracts. Redha^12^ stated that while *N. oleander* reported the presence of substances such as phenol, tannin, coumarin, alkaloid and sterol in flower tissues, negative results were obtained in terms of saponin and antroquinone glycosides.

### Quantitative phytochemical analysis

**Figure 4:**
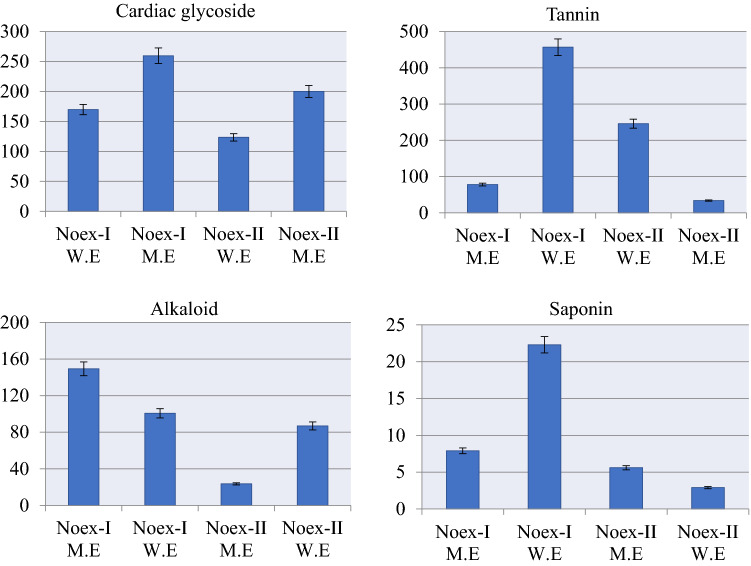
Quantitative analysis of phytochemicals in Noex-I and Noex-II. Cardiac glycoside: mgSE/g, tannin: mgTE/g, saponin mgDE/g, alkaloid: mg AE/g.

Quantitative analyzes of the phytochemical ingredients, whose presence were detected by qualitative analysis, were also carried out and the results are given in Fig. 4. Parallel results were obtained with qualitative analyzes, and it was determined that the extract obtained with water generally had a richer phytochemical content. The cardiac glycoside content in water extracts of *N. oleander* leaf and flower were 169.89 ± 0.21 and 123.44 ± 0.10 mg SE/g, respectively. In methanol extract, cardiac glycoside content was 259.71 ± 0.23 and 200.25 ± 0.31 mg SE/g for *N. oleander* leaf and flower extracts, respectively. The tannin content, which is the major phytochemical in the *N. oleander* leaf extract, is 456.82 ± 0.33 mgTE/g in the water extract and 77.90 ± 0.11 mgTE/g in the methanol extract. The amount of tannin in *N. oleander* flower extracts is 46.27% lower than *N. oleander* leaf extract. A higher amount of saponin was detected in water extracts of *N. oleander* leaf extract as 22.39 ± 0.09 mgDE/g. Saponin, which was evaluated as negative in *N. oleander* flower extract water extract in qualitative analyzes, was detected at a rate as low as 2.91 ± 0.08 mgDE/g in quantitative analyzes of *N. oleander* flower extract. The alkaloid, which is highly found in the methanol extract of *N. oleander* leaf is more concentrated in the water extract of *N. oleander* flower. While no alkaloid was detected in the methanol extract of *N. oleander* flower in qualitative analysis, this substance was detected at low rates in quantitative analysis. In the literature, quantitative phytochemical analyzes have been carried out on *Nerium* species grown in different ecological conditions. Aslam et al.^43^ reported that *Nerium indicum* collected from Panjgur, Balochistan contains 0.128 g of alkaloids, 0.249 g of saponins and 0.295 g of flavonoids. Bhuvaneshwari et al.^41^ reported that *N. oleander* samples collected from Yercaud (Tamilnadu) contained terpenoids, alkaloids, glycosides, saponins, tannins, and no flavonoids and flobatanine.

### Physiological parameters and relative injury rates

The effects of *N. oleander* leaf and flower applications on germination-related parameters and the relative injury rates are given in Table 2. *N. oleander* leaf and flower treatments caused a significant decrease in germination parameters in *A. cepa* bulbs. While 100% germination rate was determined in the bulbs of the control group, the germination percentage was between 72 and 88% in the *N. oleander* leaf extract applications at 50–250 μg/mL concentrations. The most significant decrease in germination rate was detected in the group treated with 250 μg/mL *N. oleander* leaf extract, and there was a 1.38-fold decrease in germination rate in this group compared to the control group. *N. oleander* flower extract application also decreased germination, but this regression remained at lower levels compared to *N. oleander* leaf extract. In the 250 μg/mL *N. oleander* flower extract treated group, the germination rate decreased by 1.29 times compared to the control and decreased to 77%. An increase in root length and weight ratio is observed with germination in plants. Germination rates, root elongation and weight gain increased in parallel with each other in control bulbs. The decrease in germination rates caused by *N. oleander* leaf and flower applications was also detected in root elongation and weight gain, and it was determined that these regressions increased depending on the dose. 250 μg/mL *N. oleander* leaf and flower applications caused 36.8% and 29.8% reduction in root length compared to the control group, respectively (p < 0.05). 250 μg/mL *N. oleander* leaf and flower applications caused 48.3% and 40.2% decrease in weight gain compared to the control group, respectively (p < 0.05). When the relative injury rates calculated based on the control group germination percentage were examined, the highest relative injury rate was found as 0.28 in the 250 μg/mL *N. oleander* leaf extract applied group.

**Table 2:** Effect of Noex-I and Noex-II applications on germination-related parameters.

Groups	GP (%)	Root length (cm)	Weight gain (g)	
Group I	100	8.7 ± 0.92^a^	+ 6.78^a^	
Group II	88	7.5 ± 0.86^b^	+ 5.62^b^	
Group III	81	6.6 ± 0.78^c^	+ 4.38^c^	
Group IV	72	5.5 ± 0.67^d^	+ 3.50^d^	
Group V	93	7.9 ± 0.88^b^	+ 6.00^b^	
Group VI	86	7.0 ± 0.81^c^	+ 4.99^c^	
Group VII	77	6.1 ± 0.69^d^	+ 4.05^d^	

Group I: Control, Group II: 50 μg/mL Noex-I, Group III: 100 μg/mL Noex-I, Group IV: 250 μg/mL Noex-I, Group V: 50 μg/mL Noex-II, Group VI: 100 μg/mL Noex-II, Group VII: 250 μg/mL Noex-II. Values are shown as mean ± SD. The averages shown with different letters^(a–d)^ in the same column are significant at p < 0.05. *GP* Germination percentage, *RIR* Relative injury rate.

The germination-reducing effects of *N. oleander* leaf and flower applications are closely related to the active ingredients it contains. In this study, the presence of cardiac glycosides in *N. oleander* leaf and flower extracts was determined by phytochemical analysis. Oleandrin, a cardiac glycoside, whose presence in *N. oleander* has been determined by many studies, causes disruptions in physiological and biochemical pathways in cells by changing membrane fluidity, increasing intracellular calcium, inducing reactive oxygen species production, oxidative damage and mitochondrial damage^5,44^. Alkaloids, whose presence was detected by the phytochemical analysis of *N. oleander* leaf and flower, induce cell cycle arrest in G_1_ or G_2_/M phases and cause decreases in cell proliferation through apoptosis^45^. Disruptions in physiological reactions and germination-related metabolism in *Allium* bulbs can be explained by the induction of oxidative stress and retarding effects on the cell cycle of active compounds in *N. oleander* leaf and flower extract. Although there is no study in the literature on the physiological effects of *Nerium* extracts on *A. cepa*, there are studies investigating various allelopathic effects in plants. Supporting our findings, Mojarad et al.^46^ reported that *Nerium* leaf and flower extracts caused reductions in shoot length, root length and fresh weight of seedlings of monocotyledonous *Hordeum vulgare* and dicotyledonous *Vicia sativa* seedlings. Karaaltın et al.^47^ reported that *Nerium* extracts significantly reduced root length in beans and wheat, and this reduction was associated with the allelopathic effect of the extract.

### Antioxidant/oxidant dynamics

**Figure 5:**
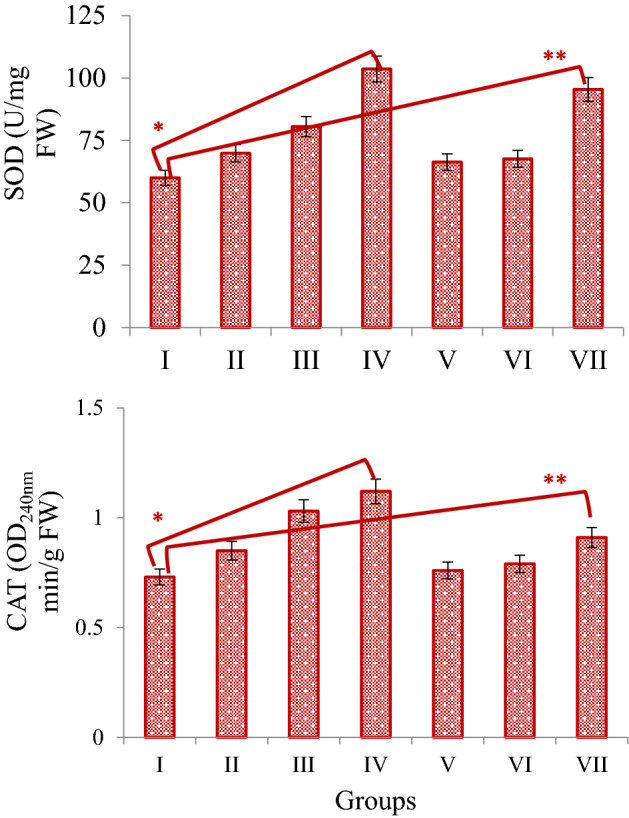
The effect of Noex-I and Noex-II applications on SOD and CAT activities. Group I: Control, Group II: 50 μg/mL Noex-I, Group III: 100 μg/mL Noex-I, Group IV: 250 μg/mL Noex-I, Group V: 50 μg/mL Noex-II, Group VI: 100 μg/mL Noex-II, Group VII: 250 μg/mL Noex-II. *Indicates the statistical difference between Group I and IV, **Indicates the statistical difference between Group I and VII (p < 0.05).

In order to determine the effects of *N. oleander* leaf and flower applications on antioxidant/oxidant dynamics, the levels of MDA, GSH, SOD and CAT in root tip cells were examined. SOD and CAT are important endogenous antioxidant enzymes and changes in the activities of these enzymes are closely related to oxidative stress in the cell. *N. oleander* leaf extract application significantly increased SOD and CAT activity compared to the control group, depending on the dose. The most significant increase was detected in Group IV administered 250 μg/mL *N. oleander* leaf extract. SOD and CAT activity increased 72.6% and 53.4%, respectively, in Group IV compared to the control group. This shows that the application of *N. oleander* leaf extract induces the SOD enzyme at a higher rate than the CAT enzyme. Similar increases in enzyme activities were observed in the application of *N. oleander* flower extract, but these increases were found to be at a lower level compared to the effects of *N. oleander* leaf extract. Especially, 50 μg/mL and 100 μg/mL *N. oleander* flower extract applications did not cause a statistically significant (p > 0.05) increase in CAT activity, while 250 μg/mL dose caused a 24.6% increase compared to the control group (p < 0.05). *N. oleander* flower application caused more significant changes in SOD activity compared to CAT enzyme. While 50 μg/mL and 100 μg/mL *N. oleander* flower extract administrations provided a similar increase in SOD activity, 250 μg/mL *N. oleander* flower extract increased SOD activity by 59% compared to the control group (Fig. 5). When all the results were evaluated together, it was determined that *N. oleander* leaf application significantly induced and increased SOD and CAT activity at a dose of 250 μg/mL. While the application of *N. oleander* flower extract did not cause a dominant change in enzyme activities, especially at doses of 50 μg/mL and 100 μg/mL, 250 μg/mL dose significantly induced SOD and CAT activities.

SOD and CAT activities are induced in the presence of oxidative stress and increased enzyme activities after *N. oleander* leaf and flower applications indicate the formation of oxidative stress in the cell. As a result of biochemical reactions such as respiration and photosynthesis and many metabolic activities in plants, free radicals such as hydroxyl radical, superoxide anion and hydrogen peroxide are formed. These radicals cause oxidative stress in the cell. The antioxidant defense system neutralizes this stress and cellular metabolism continues without interruption. As a result of various agents or stress in cells, exogenous factors increase the production of reactive oxygen species and the activities of antioxidant enzymes are also induced. SOD is an important antioxidant enzyme induced in the presence of oxidative stress and is involved in the dismutation of superoxide radicals^48,49^. The increase observed in SOD activity as a result of the application of *N. oleander* leaf and flower in this study indicates an increase in oxidative stress. An increase in SOD activity causes an increase in hydrogen peroxide levels in the cell, which induces CAT activity. CAT activity is very important for the survival of plants exposed to stress factors^50,51^. The increases in SOD and CAT activities indicate that *N. oleander* leaf and flower applications cause oxidative stress in *A. cepa* root tip cells.

**Figure 6:**
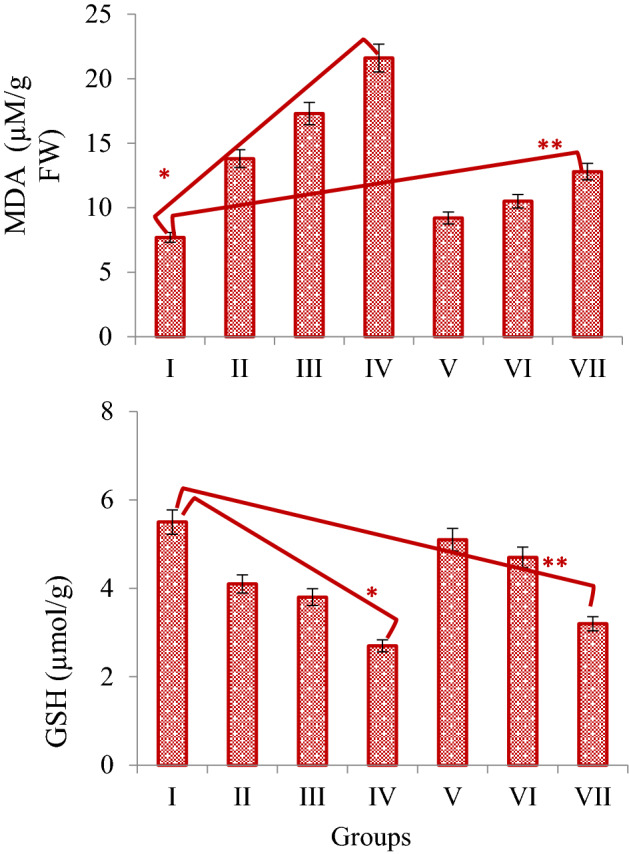
The effect of Noex-I and Noex-II applications on MDA and GSH levels. Group I: Control, Group II: 50 μg/mL Noex-I, Group III: 100 μg/mL Noex-I, Group IV: 250 μg/mL Noex-I, Group V: 50 μg/mL Noex-II, Group VI: 100 μg/mL Noex-II, Group VII: 250 μg/mL Noex-II. *Indicates the statistical difference between Group I and IV, **Indicates the statistical difference between Group I and VII (p < 0.05).

In the *N. oleander* leaf and flower applied groups, dose-dependent increases were also observed in the MDA levels, with the most significant increase detected in the 250 μg/mL dose of both applications. 250 μg/mL *N. oleander* leaf and flower applications increased MDA levels in root tip cells by 180.5% and 66.2%, respectively, compared to the control group. Along with the increase in MDA levels, there was a decrease in GSH levels, which is an endogenous and powerful antioxidant. 250 μg/mL *N. oleander* leaf and flower applications decreased GSH levels in root tip cells by 50.9% and 41.8%, respectively, compared to the control group (Fig. 6). The decrease in GSH levels and increase in MDA levels show that *N. oleander* leaf and flower applications cause deterioration in antioxidant-oxidant dynamics. MDA is produced at low levels in cells under normal conditions and is used in different pathways such as signal transduction and gene expression. Increases in MDA production are observed in cells under stress conditions and in the presence of oxidative stress. Increases observed in MDA level in a cell indicate oxidative stress and lipid peroxidation that develops accordingly^52^. The fact that *N. oleander* leaf and flower applications increase the MDA level indicates that oxidative stress is induced. Along with the increase in MDA, a decrease in GSH levels in root tip cells was also determined. GSH is a powerful antioxidant molecule found in various organelles in plants and scavenging free radicals. The decrease in GSH levels indicates that oxidative stress in the cell increases and the reduced-GSH is rapidly oxidized^53^. As a result of *N. oleander* leaf and flower applications, the increase in MDA and the decrease in GSH levels indicate that the antioxidant/oxidant balance in the cell is impaired. Although there is no information in the literature that *N. oleander* applications induce oxidative stress in *A. cepa*, there are studies reporting that it causes oxidative damage in many cell types. Atroshi et al.^54^ found that *N. oleander* induced oxidative damage, Sreenivasan et al.^55^ reported that it increased oxidative stress by triggering the formation of reactive intermediates. Calderón-Montaño et al.^56^ reported that *N. oleander* extract exhibited cytotoxic activity by inducing the formation of reactive oxygen species in cells.

### Cytotoxic effects

**Figure 7:**
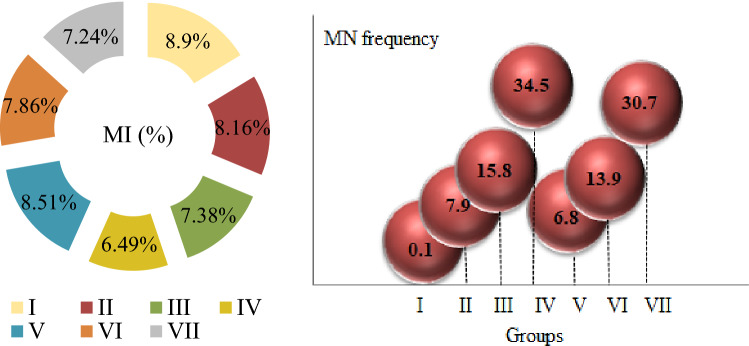
The effects of Noex-I and Noex-II applications on MI(%) and MN frequencies. Group I: Control, Group II: 50 μg/mL Noex-I, Group III: 100 μg/mL Noex-I, Group IV: 250 μg/mL Noex-I, Group V: 50 μg/mL Noex-II, Group VI: 100 μg/mL Noex-II, Group VII: 250 μg/mL Noex-II.

The cytotoxic effects of *N. oleander* leaf and flower applications on *A. cepa* root tip cells were determined by examining the MN frequencies and MI rates, and the results are given in Fig. 7. MI is an indicator of cell proliferation and it was determined that 890 ± 22.5 cells were divided among the total cells counted in the control group. Both *N. oleander* leaf and flower applications caused a decrease in the number of dividing cells, resulting in a decrease in MI rates. This regression increased depending on the dose, and the most prominent cytotoxic effect occurred at 250 μg/mL doses of both extracts. *N. oleander* leaf and flower applications at the highest dose tested in this study caused 1.37 and 1.22-fold reductions in the number of dividing cells, respectively. It was also found that the reductions observed at all doses tested were statistically significant (p < 0.05). Reductions in the number of dividing cells were also reflected in the MI rates, and MI rates were calculated as 8.9%, 6.46%, and 7.24% in the control, 250 µg/mL *N. oleander* leaf extract, and 250 µg/mL *N. oleander* flower-treated groups, respectively. MI is a reliable indicator for evaluating cell proliferation and determining the cytotoxic effects of various agents. The cytotoxic effects of compounds in living cells can occur by many mechanisms. Inhibition of microtubule formation and deformation of spindle fibers cause delays or interruptions in cell division, resulting in regressions in MI rates. Such damages also cause different types of abnormalities such as c-mitosis, multipolar anaphase, MN and sticky chromosomes in the advanced stages of division. As a result of these abnormalities, reductions of 50% or more in the MI rates of cells indicate a lethal cytotoxic effect, while reductions of 50% or less indicate a sublethal effect^57^. According to this distinction, *N. oleander* leaf and flower extract applications caused cytotoxicity by exhibiting a sub-lethal effect on *A. cepa* root tip cells. Cytotoxic agents cause various changes in cells such as multipolar anaphases, multinucleated cells, and vagrant chromosomes that induce MN formation by disrupting the spindle organization. All these changes cause decreases in MI rates and increases in MN frequencies. Similarly, there are studies in the literature reporting the effect of *Nerium* extracts on cell proliferation. Al-Razzaq^58^ reported that *Nerium* extracts caused a 78% regression in MI by reducing cell proliferation. Another parameter used to determine the cytotoxic effect is the frequency of MN. MN formations are a sign of cytotoxic and genotoxic effects in cells. The aneugenic effect, which occurs as a result of damage to the spindle fibers during cell division, also causes an increase in MN frequencies. In this study, while a negligible MN formation was detected in the control group (p > 0.05), statistically significant MN frequencies were determined in all groups treated with *N. oleander* leaf and flower extract (p < 0.05). MN frequencies were detected as 7.90 ± 0.87, 15.8 ± 1.46 and 34.5 ± 2.74 in 50 μg/mL, 100 μg/mL and 250 μg/mL *N. oleander* leaf extract treated groups, respectively. In the groups administered 50–250 μg/mL *N. oleander* flower extract, the MN frequency was counted in the range of 6.80 ± 0.85–30.7 ± 2.64. MN formations originate from spindle damage, chromosome breaks or lagging chromosomes in cells. In the process of new nucleus formation, the laggard chromosomes condense before fully integrated into the nucleus during telophase, forming a nuclear envelope, transforming into a nuclear bud and then MN. Spindle thread abnormalities in particular are important triggers of MN formation. Abnormalities occurring in the G_1_ phase of the cell cycle reveal MN formations in the late stages of division^59,60^. Decreases in MI rates detected in *N. oleander* leaf and flower extract treated groups also support the increase in MN frequency. The results obtained from MI and MN analyzes indicate that both extracts exhibit cytotoxic effects by reducing cell proliferation and increasing MN frequencies and that the cytotoxic effect of *N. oleander* leaf extract is higher than *N. oleander* flower extract. Cardiac glycosides are potent inhibitors of cell division. Cardiac glycosides significantly affect cell proliferation by inhibiting the pumps in the cell membrane, especially Na–K-ATPase^61^. In the phytochemical analyzes performed in this study, *N. oleander* leaf and flower extracts were found to contain cardiac glycosides, and the decrease in MI rates and the increase in MN frequencies observed in *A. cepa* root tip cells were associated with these active compounds. Similarly, Tarkowska^62^ reported that oleandrine glycosides exhibit antimitotic activity and cause MN formation.

### Genotoxic effects

**Figure 8:**
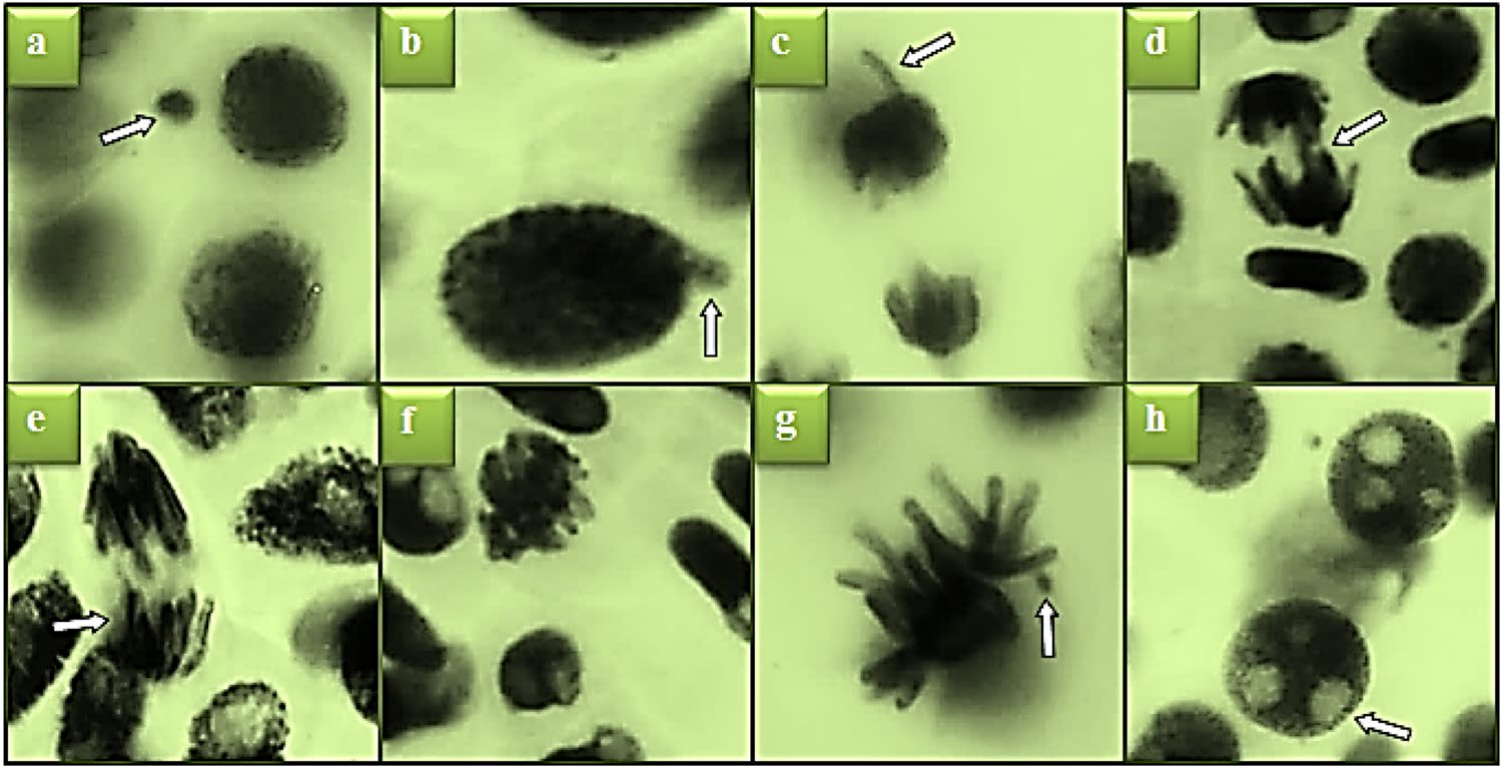
Appearances of MN and CAs induced by Noex-I. MN (**a**), nuclear bud (**b**), vagrant chromosome (**c**), bridge (**d**), unequal distribution of chromatin (**e**), sticky chromosome (**f**), fragment (**g**), vacuolated nucleus (**h**).

**Figure 9:**
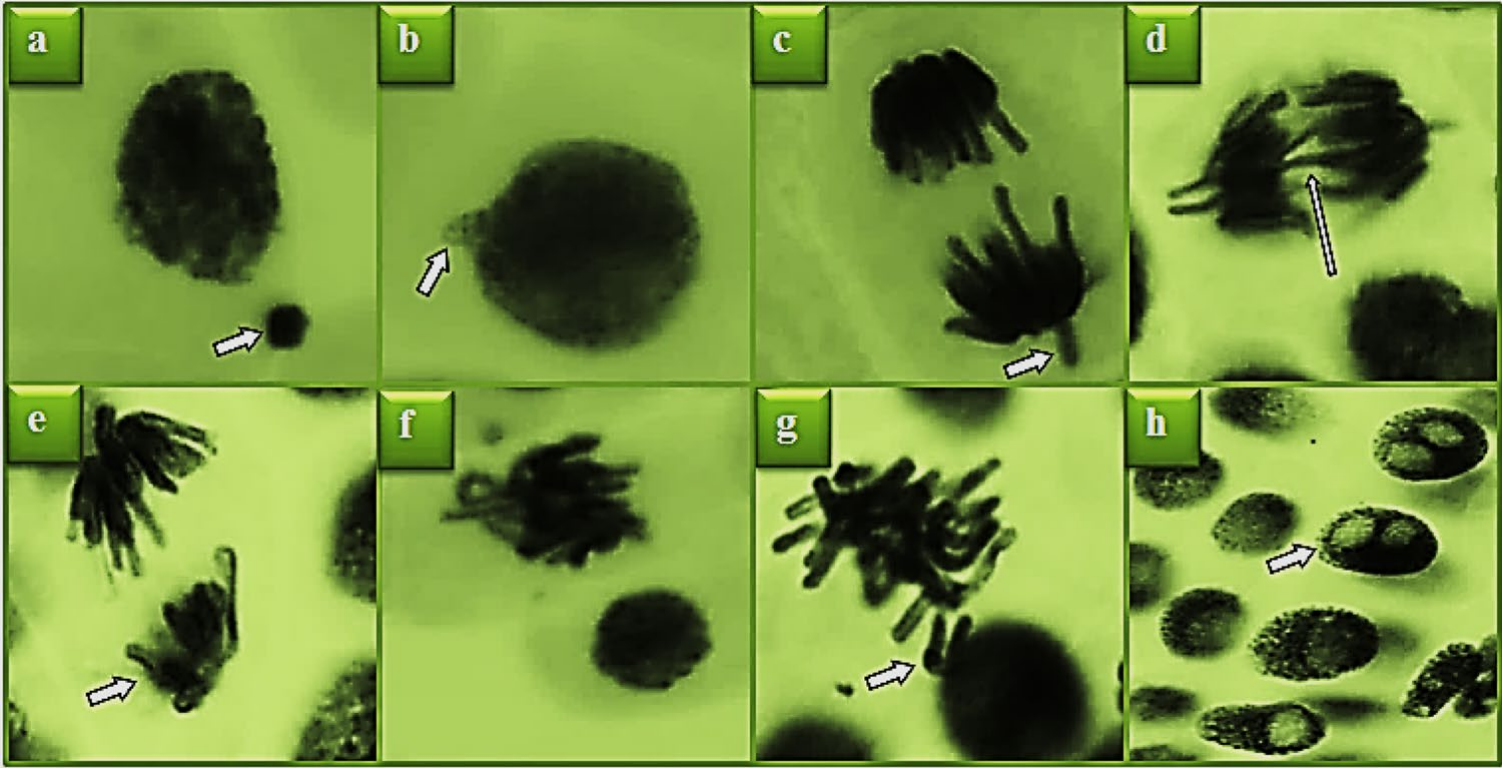
Appearances of MN and CAs induced by Noex-II. MN (**a**), nuclear bud (**b**), vagrant chromosome (**c**), bridge (**d**), unequal distribution of chromatin (**e**), sticky chromosome (**f**), fragment (**g**), vacuolated nucleus (**h**).

Possible genotoxic effects of *N. oleander* leaf and flower applications on *A. cepa* root tip cells were determined by CAs test. In the control group, statistically insignificant abnormality of sticky chromosome and unequal distribution of chromatin were detected (p < 0.05), while high rates of different types of CA formations were observed in *N. oleander* leaf and flower applications (Table 3). Nuclear bud, vagrant chromosome, bridge, unequal distribution of chromatin, sticky chromosome, fragment and vacuolated nucleus are common CAs observed in both applications (Figs. 8, 9). The frequency of CAs increased with the increase of the extract dose, and the highest frequencies were found in Group IV and Group VII, where 250 μg/mL *N. oleander* leaf and flower extracts were administered, respectively. *N. oleander* leaf application caused the highest frequency of sticky chromosome formations among CAs, and *N. oleander* flower application caused the highest frequency of nuclear bud formation.

**Table 3:** CAs frequencies induced by Noex-I and Noex-II applications.

CAs	Group I	Group II	Group III	Group IV	Group V	Group VI	Group VII
NB	0.00 ± 0.00^d^	6.60 ± 0.80^c^	9.90 ± 1.33^b^	27.3 ± 2.36^a^	6.30 ± 0.80^c^	10.5 ± 1.30^b^	27.8 ± 2.53^a^
VC	0.00 ± 0.00f.	3.90 ± 0.65^e^	8.50 ± 0.93^c^	13.5 ± 1.38^b^	5.20 ± 0.76^d^	8.80 ± 1.18^c^	24.6 ± 2.33^a^
B	0.00 ± 0.00^d^	4.90 ± 0.74^c^	7.20 ± 1.18^b^	16.4 ± 1.52^a^	4.00 ± 0.69^c^	6.10 ± 1.02^b^	15.1 ± 1.42^a^
UDC	0.20 ± 0.42^e^	4.20 ± 0.69^d^	7.00 ± 1.05^c^	15.2 ± 1.44^a^	3.50 ± 0.58^d^	4.20 ± 0.89^d^	13.8 ± 1.34^b^
SC	0.30 ± 0.48f.	5.10 ± 0.85^d^	16.0 ± 1.42^b^	31.8 ± 2.61^a^	3.00 ± 0.53^e^	5.80 ± 0.84^d^	12.9 ± 1.25^c^
FRG	0.00 ± 0.00f.	3.60 ± 0.62^e^	9.00 ± 0.88^c^	12.9 ± 1.35^a^	2.80 ± 0.50^e^	6.80 ± 0.77^d^	10.8 ± 1.16^b^
VN	0.00 ± 0.00f.	3.30 ± 0.59^d^	5.40 ± 0.83^c^	11.7 ± 1.29^a^	2.50 ± 0.46^e^	3.60 ± 0.75^d^	9.20 ± 1.04^b^

For CAs analysis, 1000 cells were analyzed in each group. The averages shown with different letters^(a–f)^ in the same line are significant at p < 0.05. *NB* nuclear bud, *VC* vagrant chromosome, *B* bridge, *UDC* unequal distribution of chromatin, *SC* sticky chromosome, *FRG* fragment, *VN* vacuolated nucleus.

Chromosomal condensations, DNA depolymerization, and dissolved nucleoproteins induce stickiness in chromosomes. It is also reported in literature studies that sticky chromosome formations cause lethal effects in cells^63,64^. The formation of highly sticky chromosomes as a result of *N. oleander* leaf administration also supports the reduction in MI rates, and the reduction in cell proliferation is mainly due to irreversible irregularities in mitosis, including stickness. Nuclear bud, which was detected with the highest rate among abnormalities in *N. oleander* flower application, is closely related to MN formations. High MN frequencies observed as a result of *N. oleander* leaf and flower applications confirm the nuclear bud and MN relationship. Both nuclear buds and MN are of an aneuploidogenic origin. Nuclear buds separate from the nucleus in the later stages of division to form an MN, and in some cases the buds re-integrate into the nucleus. Nuclear buds also cause chromatin bridges or different chromosomal rearrangements observed in abnormal anaphases. Nuclear buds observed in both extract applications also induce other CAs. Many active ingredients detected in *N. oleander* leaf and flower extracts may cause CAs by exhibiting genotoxic effects. Cardiac glycosides detected in *Nerium* flower and leaf extracts have an important place in the genotoxic effect. Cardiac glycosides are potent inhibitors of repair of DNA double-strand breaks^65^. Alkaloids, which are among the other active compounds found in the extracts, exhibit genotoxic effects by causing cross-linking and breaks in DNA, sister chromatid changes and the formation of chromosome mutations^66^. MDA resulting from lipid peroxidation is another possible cause of CAs formations induced by *N. oleander* leaf and flower applications. In antioxidant/oxidant dynamic studies, it was determined that both extracts increased intracellular MDA levels. MDA has a highly electrophilic property and this reactivity causes MDA to bind to macromolecules such as protein and DNA, then cause damages^52,67^. MDA causes structural abnormalities by triggering intermolecular and intramolecular cross-linkings in the DNA structure. The fact that *N. oleander* leaf and flower extracts both increase MDA level by inducing oxidative stress and exhibit genotoxic effects by the active ingredients in their content explain the formation of different types of CAs. Similarly, Calderón-Montaño et al.^56^ determined that *N. oleander* extract exhibited a cytotoxic effect by causing DNA damage in various cell lines.

### Anatomical alterations

**Figure 10:**
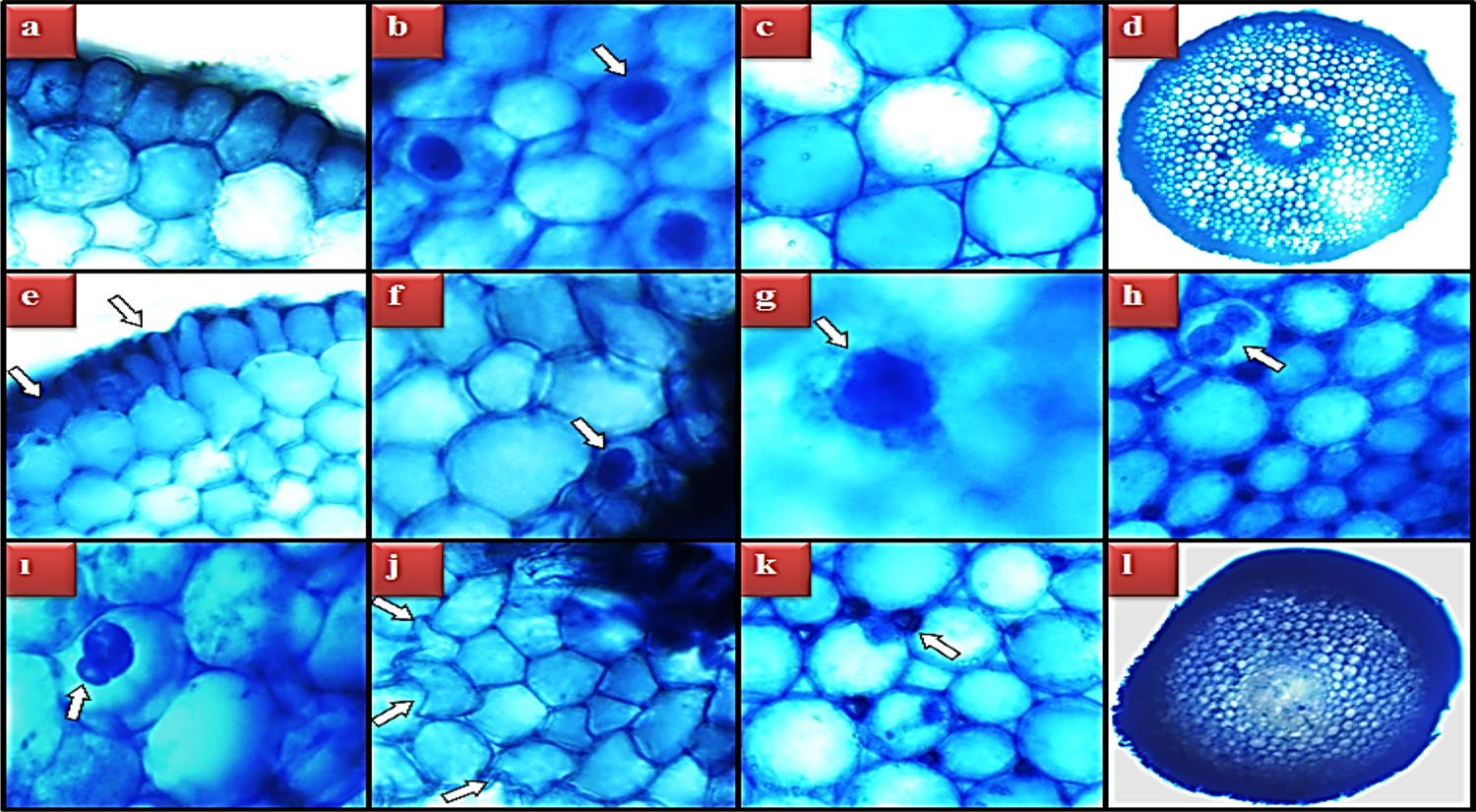
Anatomical changes induced by Noex-I. Normal appearance of epidermis cells (**a**), normal appearance of cell nucleus-*oval* (**b**), normal appearance of cortex cells (**c**), normal appearance of vascular tissue (**d**), epidermis cell damage (**e**), flattened cell nucleus (**f**), giant cell nucleus (**g**), binuclear cell (**h**), cell with MN (**i**), cortex cell damage (**j**), cortex cell wall thickening (**k**), indistinct vascular tissue (**i**).

**Figure 11:**
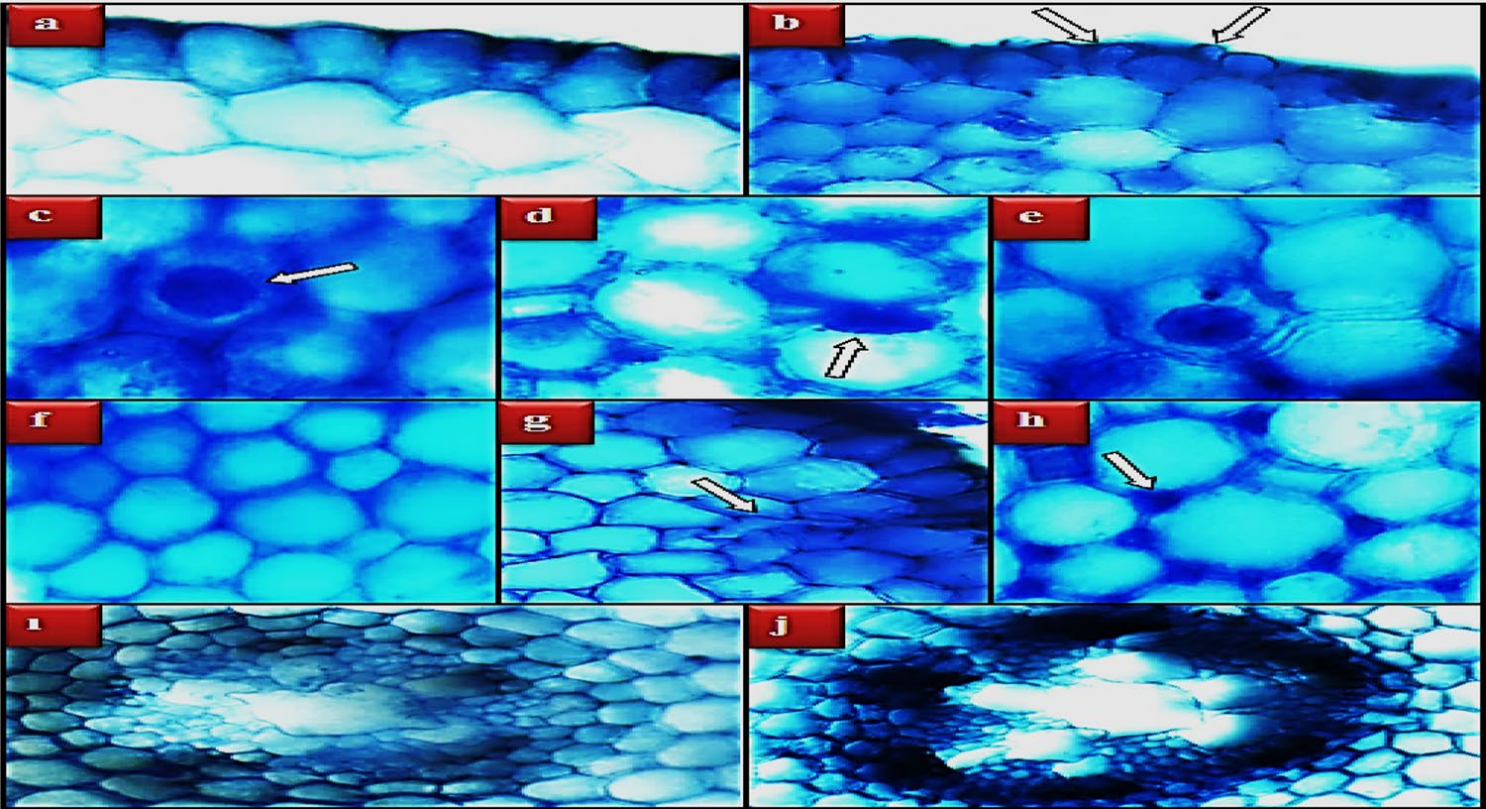
Anatomical changes induced by Noex-II. Normal appearance of epidermis cells (**a**), epidermis cell damage (**b**), normal appearance of cell nucleus-*oval* (**c**), flattened cell nucleus (**d**), cell with MN (**e**), normal appearance of cortex cells (**f**), cortex cell damage (**g**), thickening of the cortex cell wall (**h**), normal appearance of vascular tissue (**i**), indistinct vascular tissue (**j**).

Anatomical changes induced by *N. oleander* leaf and flower applications were investigated by examining the cross-sections of the root tip of *A. cepa*. After the application of *N. oleander* leaf, epidermis cell damage, flattened cell nucleus, giant nucleus, binuclear cell, cortex cell damage, thickening of the cortex cell wall, and indistinct conduction tissue damage were detected in the root tip anatomy (Fig. 10). In the 250 μg/mL *N. oleander* leaf applied group, epidermis cell damage, flattened cell nucleus, cortex cell damage and thickening of the cortex cell wall were very severe; giant nucleus, binuclear cell, and indistinct conduction tissue were found to be among the low-level damages (Table 4). Anatomical changes in *N. oleander* flower applied groups occurred at a lower level compared to *N. oleander* leaf (Fig. 11). Giant nucleus and binuclear cell damage were not detected in the *N. oleander* flower administration groups. In the group administered 250 μg/mL *N. oleander* flower, epidermis cell damage, flattened cell nucleus, cortex cell damage and thickening of the cortex cell wall were observed at a moderate frequency, while indistinct vascular tissue was observed at a low frequency (Table 4). These changes in the anatomical structure are defense reactions developed against external agents and aimed at protecting the internal tissues. Thickening of the cortex cell wall occurs as a result of the accumulation of substances such as lignin, cellulose, suberin, and cutin. In this way, the thickening and durability of the cortex cell wall are increased, limiting the entry of various agents, and preventing the access of toxic compounds to the central cylinder and spreading to other tissues^36,68^. Increased expression of lignin-related genes has been reported in plants contaminated with harmful chemicals and pathogens^69,70^. Root cells can increase the number of epidermis cells to prevent exogenous agents from entering the cell. This increase causes deformations and epidermis cell damage by increasing the pressure of the cells on each other. Another anatomical change observed in the study is the flattening of the cell nucleus. The flattened nucleus arises as a result of changes in the physiological, biochemical and DNA integrity of the cell nucleus. The cell nucleus is typically spherical or elliptical in nature, but shape change can occur in response to physical or environmental changes^71,72^. All these changes observed in the anatomical structure of the groups treated with *N. oleander* leaf and flowers emerge as a result of cumulative effects. There are similar studies in the literature reporting that *Nerium* extracts cause changes in plant anatomy. Mojarad et al.^33^ reported that *Nerium* extracts significantly increased the root diameter and number of veins in monocotyledonous and dicotyledonous plants compared to the control group.

**Table 4:** Severity of anatomical alterations induced by Noex-I and Noex-II applications.

Groups	ECD	FCN	GCN	BN	CCD	CCWT	IVT
Group I	**−**	**−**	**−**	**−**	**−**	**−**	**−**
Group II	**+**	**+**	**−**	**−**	**+**	**+**	**−**
Group III	**++**	**++**	**−**	**−**	**++**	**++**	**−**
Group IV	**+++**	**+++**	**+**	**+**	**+++**	**+++**	**+**
Group V	**−**	**−**	**−**	**−**	**−**	**−**	**−**
Group VI	**+**	**+**	**−**	**−**	**+**	**+**	**−**
Group VII	**++**	**++**	**−**	**−**	**++**	**++**	**+**

*ECD* epidermis cell damage, *FCN* flattened cell nucleus, *GCN* giant cell nucleus, *BN* binuclear cell, *CCD* cortex cell damage, *CCWT* cortex cell wall thickening, *IVT* indistinct vascular tissue. (−): no damage, ( +): little damage, (++): moderate damage, (+++): severe damage.

## Conclusion

Many parts of plants can have toxic effects due to the secondary metabolites they contain. Therefore, the potential toxicity of plants commonly used in homes, gardens or landscapes poses a significant risk. It is of great importance to investigate the potential toxicities of plants as well as their protective properties. In the literature, mostly the protective properties of herbal extracts were investigated, and studies on their toxic effects were insufficient. Plant phytochemicals have an important role in the formation of toxicity. Therefore, growing a plant of the same species in different ecological conditions may cause changes in its toxicity profile. In this respect, the fact that the plant diversity in the world is very wide and the phytochemical content of the same plant changes according to the ecological conditions makes the toxicity studies of herbal extracts insufficient. In this study, the toxicity profile and qualitative/quantitative phytochemical analyzes of the flower and leaf parts of the *N. oleander* collected from Giresun (Turkiye) were investigated. Toxicity studies were investigated using *A. cepa*, a bioindicator organism, and in this way, possible potential effects in eukaryotes and allelopathic effects of extracts were investigated. Flower and leaf extracts caused regressions in *Allium* germination and development, and these regressions were thought to be related to oxidative stress in the plant. The fact that extract applications cause changes in antioxidant enzyme levels, increase in MDA levels and decrease in GSH levels confirms oxidative stress. The extracts, which exhibited genotoxic effect by causing higher CAs formation, also showed cytotoxic effects by reducing MI rates and increasing MN frequencies. The toxic effects of leaf and flower extracts can be associated with the active ingredients they contain, and as a result of phytochemical analysis, it was determined that the leaf and flower extracts contained cardiac glycosides, saponins, tannins and alkaloids. Toxicity of both flower and leaf extract may occur as a result of the cumulative action of all active ingredients and may be associated with more than one mechanism. The toxicity of the leaf extract is higher than the flower extract, which can be explained by the higher concentration of the phytochemicals tested in the leaf. The toxic effects of plants used in the garden, home, office and all landscaping applications should not be ignored and reliable environments should be created without using toxic plants if possible. This study, in which the toxic effects of *N. oleander* leaf and flower extracts were determined, will lead to the investigation of the toxicity profiles of plants due to their phytochemical content.

## Supplementary Information

Supplementary Information.

## Data Availability

The datasets used and/or analyzed during the current study are available from the corresponding author on reasonable request.
