# Supplementary figures and images for: Qualitative and quantitative phytochemical screening of Nerium oleander L. extracts associated with toxicity profile
Source: Sci Rep. 2022 Dec 11;12:21421. doi: 10.1038/s41598-022-26087-0 (PMC9742154; doi:10.1038/s41598-022-26087-0)

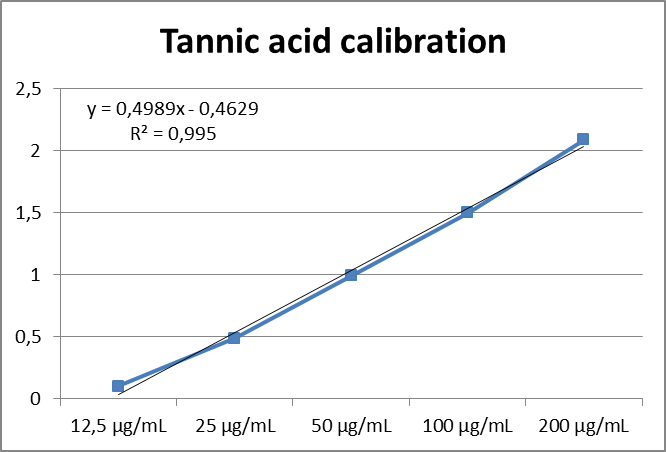

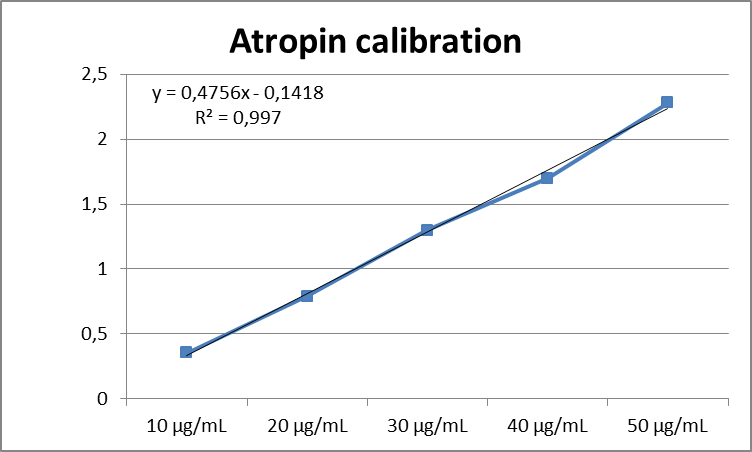


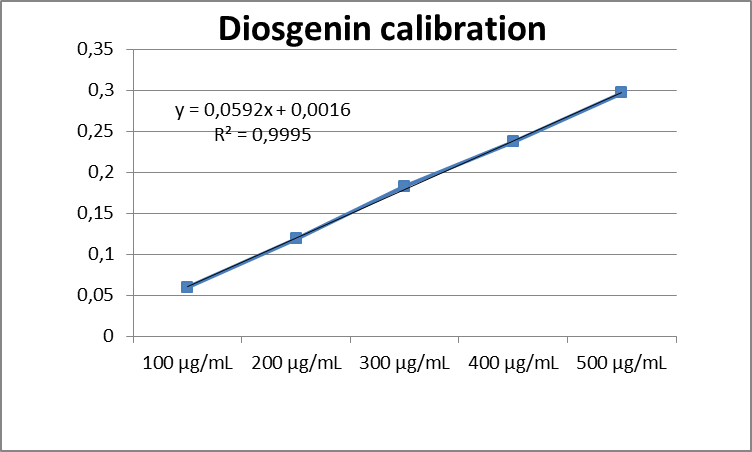

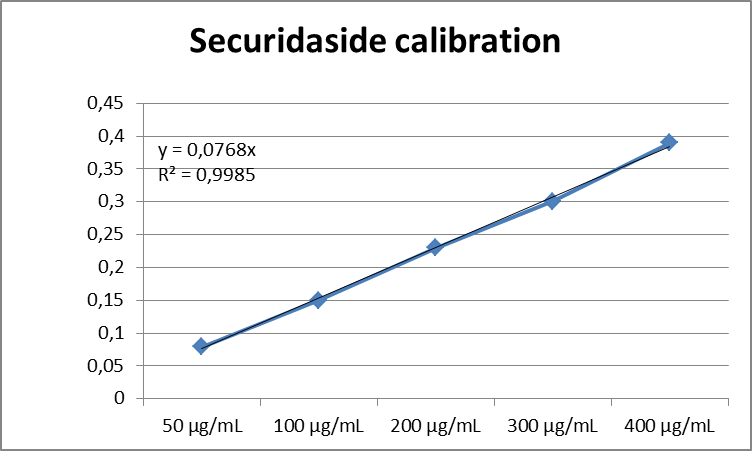


Calibration curves of phytocemical substances

Supplement: Supplementary file 1 — Supplementary Information. [file 41598_2022_26087_MOESM1_ESM.docx]
